# Payoffs, Not Tradeoffs, in the Adaptation of a Virus to Ostensibly Conflicting Selective Pressures

**DOI:** 10.1371/journal.pgen.1004611

**Published:** 2014-10-02

**Authors:** Lindsey W. McGee, Erick W. Aitchison, S. Brian Caudle, Anneliese J. Morrison, Lianqing Zheng, Wei Yang, Darin R. Rokyta

**Affiliations:** 1 Department of Biological Science, Florida State University, Tallahassee, Florida, United States of America; 2 Institute of Molecular Biophysics, Florida State University, Tallahassee, Florida, United States of America; 3 Department of Chemistry and Biochemistry, Florida State University, Tallahassee, Florida, United States of America; University of Arizona, United States of America

## Abstract

The genetic architecture of many phenotypic traits is such that genes often contribute to multiple traits, and mutations in these genes can therefore affect multiple phenotypes. These pleiotropic interactions often manifest as tradeoffs between traits where improvement in one property entails a cost in another. The life cycles of many pathogens include periods of growth within a host punctuated with transmission events, such as passage through a digestive tract or a passive stage of exposure in the environment. Populations exposed to such fluctuating selective pressures are expected to acquire mutations showing tradeoffs between reproduction within and survival outside of a host. We selected for individual mutations under fluctuating selective pressures for a ssDNA microvirid bacteriophage by alternating selection for increased growth rate with selection on biophysical properties of the phage capsid in high-temperature or low-pH conditions. Surprisingly, none of the seven unique mutations identified showed a pleiotropic cost; they all improved both growth rate and pH or temperature stability, suggesting that single mutations even in a simple genetic system can simultaneously improve two distinct traits. Selection on growth rate alone revealed tradeoffs, but some mutations still benefited both traits. Tradeoffs were therefore prevalent when selection acted on a single trait, but payoffs resulted when multiple traits were selected for simultaneously. We employed a molecular-dynamics simulation method to determine the mechanisms underlying beneficial effects for three heat-shock mutations. All three mutations significantly enhanced the affinities of protein-protein interfacial bindings, thereby improving capsid stability. The ancestral residues at the mutation sites did not contribute to protein-protein interfacial binding, indicating that these sites acquired a new function. Computational models, such as those used here, may be used in future work not only as predictive tools for mutational effects on protein stability but, ultimately, for evolution.

## Author Summary

One of the most fundamental tradeoffs in evolutionary biology is between survival and reproduction. Many parasites experience distinct selective pressures during different stages of their life cycles; mutations arising during one stage may be beneficial, but come at a cost during another. For example, many viruses experience favorable growth conditions within a host punctuated with harsh conditions outside the host during transmission. We conducted an evolution experiment with a ssDNA microvirid bacteriophage selecting for growth within the host and capsid stability outside the host in the presence of extreme environmental conditions (low pH or high temperature), and we hypothesized detection of a tradeoff between reproduction and survival. We found that individual mutations gained under rapidly fluctuating selective pressures similar to those experienced by pathogens increased both growth rate and capsid stability; tradeoffs were completely absent. We compared the effects of beneficial mutations gained in response to selection for growth rate alone and found the expected tradeoffs on capsid stability. Tradeoffs therefore arise when selection is not working to avoid them. Otherwise, payoffs prevail.

## Introduction

The relationship between genotype and phenotype is complicated by genetic interactions such as pleiotropy, in which genes contribute to multiple traits thereby influencing multiple phenotypes [1], [2]. Such pleiotropic effects of mutations often manifest as tradeoffs between traits where improvement in one property entails a cost in another. This phenomenon, called antagonistic pleiotropy, is commonly observed in evolution experiments [3], [4], and genes contributing to antagonistically pleiotropic properties have been identified in various systems [4]–[6]. Antagonistic pleiotropy has been studied almost exclusively by measuring traits that were not under selection in the original experiment [4], [7]–[9]. For example, Cooper and Lenski [4] found loss of unused catabolic functions in *Escherichia coli* populations after 20,000 generations of propagation with glucose as the sole carbon source. Antagonistic pleiotropy has also been implicated in the evolution of senescence, in which pleiotropic alleles that increase performance early in life, but decrease performance later in life, accumulate in a population due to a tradeoff between early- and late-life fitness [10]–[12]. Antagonistic pleiotropy also influences the evolution of niche width by imposing costs on the evolution of particular phenotypes [13]–[16]. For example, a pathogen's specificity for its current host can minimize the risk of expansion to novel hosts resulting in a constrained host range [14], [16], [17]. Viral systems in particular may experience antagonistic pleiotropy because of their small genome sizes. The few encoded proteins must accomplish the entirety of the life cycle, which often means that some proteins are highly multifunctional. Furthermore, many viral systems, such as bacteriophages, contain overlapping genes with portions of the same nucleotide sequence encoding different proteins, allowing an individual mutation to show pleiotropy by affecting multiple proteins [18]. A single mutation with pleiotropic properties can affect either the same multifunctional protein or different proteins making it extremely difficult to optimize one trait without jeopardizing another.

The life cycle of many parasites includes periods of growth within a host punctuated with transmission events, such as passage through a digestive tract or a passive stage of exposure outside the host [19], [20]. Many parasites must survive under potentially harsh conditions between infections, and a higher proportion of the population capable of surviving the bottleneck during exposure to extreme environmental conditions may favor the evolution of increased virulence [21], [22]. Exposure to changes in environmental conditions and fluctuating selective pressures may result in tradeoffs between growth rate within a host and decay rate outside a host [23]. For example, mutations may arise that reduce decay rate during harsh conditions by increasing capsid stability, but may come at a cost to growth rate during favorable conditions by interfering with assembly kinetics. The scenario where individuals must survive outside their hosts between infection events has been explored theoretically in the context of transmission dynamics for pathogens [19], [20], and builds on earlier work with fluctuating population sizes [24] and population bottlenecks [25]–[27]. However, the impact of pleiotropic mutations on protein structure resulting in fitness tradeoffs is rarely determined.

Recent theoretical work has begun to incorporate aspects of biophysics into molecular-evolutionary models [28], and biophysical properties such as protein stability can clearly influence the evolutionary trajectory a population may take. Increased protein stability is often assumed to come at a pleiotropic cost of reduced function of the protein [28]. In viral systems, stability can have profound effects on capsid assembly kinetics [29]. The assembly reaction occurs rapidly, and the range of optimal association energies between subunits is extremely narrow. For instance, if the contact energy between subunits is too strong, the process will fall into a kinetic trap resulting in many partially formed capsids and few free subunits available to complete assembly of a full viral capsid. If the contact energy between subunits is too weak, subunits will not assemble. Assembly reactions with ideal, intermediate association energy result in almost entirely fully assembled capsids and a few free subunits with little or no intermediates [29]–[32]. Any mutation that affects the protein stability of the virus capsid may alter the association energy between proteins thereby reducing assembly efficiency, and ultimately, decreasing fitness.

**Figure 1:**
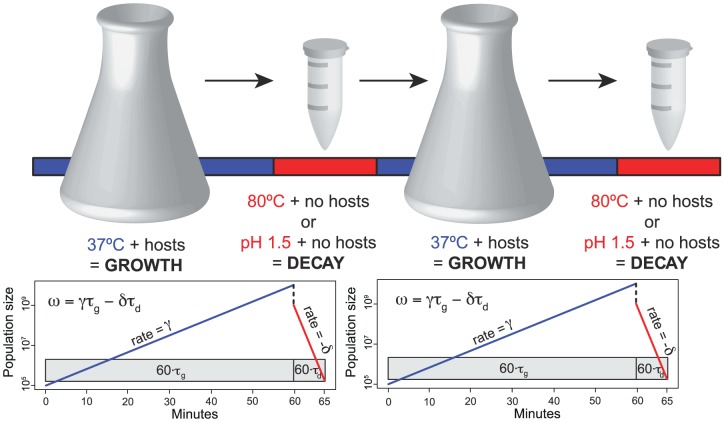
A schematic overview of our two-stage selection protocol. Two complete cycles are shown. Exponential growth at rate 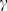 occurred for time 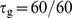 at 37°C, lasting approximately four generations. A fraction of the population was then subjected to heat-shock at 80°C or pH 1.5 for a time 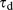 and declined at rate 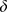. Rates were measured in doublings per hour, and times were measured as a fraction of an hour. For heat-shock selection, 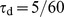; for low-pH selection, 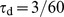. Overall fitness 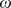 can be improved by an increase in the growth rate 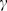 and/or a decrease in the decay rate 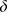. The first mutation fixed in all lineages within 11 cycles.

We investigated the relationship between viral capsid stability and growth rate under a two-stage selection regime where selection acted on both traits simultaneously. We selected for individual mutations under rapidly fluctuating selective pressures for ssDNA microvirid bacteriophages by alternating selection for increased growth rates with selection on biophysical properties of the phage capsid in the absence of growth. To do so, we developed a two-stage selection scheme (Figure 1) that naturally induces a two-component fitness consisting of a growth rate and a decay rate. Selection for increased growth rate, 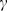, occurs during the growth phase of each transfer which lasts for time 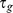. During the growth phase, the phage are allowed to replicate with an excess of hosts for approximately four generations, and the phenotype selected upon is complex; 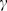 can be increased through a variety of ways (e.g., assembly rate, attachment rate, lysis time). The second stage of the regime exerts pressure upon the stability of mature viral particles. In the absence of host organisms, viral particles decay at rate 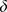 for a time, 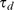, under harsh conditions until permissive hosts are present again. During this second stage of the regime, we subjected virus populations to either extreme heat (80°C) or low pH (1.5). Fitness, 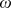, is measured as a combination of the growth and decay rates and the time spent under each condition, 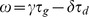, following the notation laid out by Handel et al. [20]. Benefits to survival in extreme environments may be detrimental to the replicative process by potentially disrupting the weak molecular interactions required for proper particle polymerization through over-stabilization of the protein-protein interfaces.

## Results and Discussion

### Heat-shock and pH-shock mutations come with payoffs, not tradeoffs

**Figure 2:**
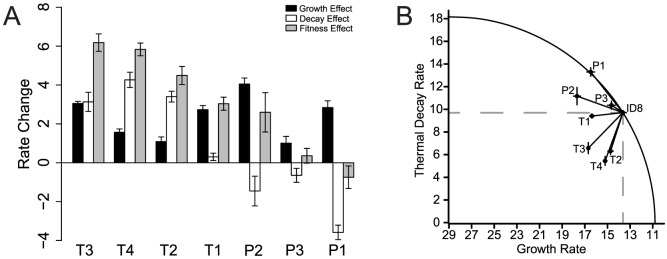
Thermal-stability and growth-rate effects of all seven mutations. We measured the effects of beneficial mutations gained under a two-stage selection scheme that naturally induces a two-component fitness, which consists of a growth rate and a decay rate (stability). (A) For visual clarity, we plotted 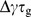, 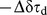, and 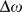 for growth, decay, and fitness effects. Overall, under the heat-shock conditions, the four unique heat-shock mutations improved growth rate, decay rate, and overall fitness relative to the ancestor genotype, ID8. The three unique low-pH mutations improved growth rate but worsened decay rate relative to the ancestor. (B) The circle plots assume an optimal decay rate of zero and an optimal growth rate of 29 (the highest we have ever seen for this family of phages) [39]. Inside the curve is beneficial overall. Inside the dotted lines is beneficial for both traits. All of the heat-shock mutations were beneficial for both traits relative to the ancestor under heat-shock conditions, but the effect for T1 was nonsignificant for 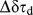.

To determine whether fluctuating selective pressures entail pleiotropic costs that may hinder adaptation, we selected for individual mutations under rapidly fluctuating selective pressures for ssDNA microvirid bacteriophages. Five lineages from the same ancestral genotype (ID8) were adapted to alternating selection for increased growth rates and selection for thermal tolerance to 80°C in the absence of growth until a single mutation fixed in the population (Figure 1), which fixed within 11 transfers. We employed full-genome sequencing for each population to identify the first fixed mutation, and plaque isolates from the evolved populations were sequence-confirmed to ensure that only a single mutation was tested. Four unique, first-step heat-shock mutations were gained in response to exposure to extreme heat-shock (Table 1). One mutation, T4, was observed in two replicate lineages. Two mutations (T1 and T2) were located in the F gene encoding for the viral coat protein, and two mutations (T3 and T4) were located in the G gene encoding for the spike protein (Table 1). Mutations in the F gene have been found to alter host range [33], and mutations in the protein G, the major spike protein, may be involved in binding the host lipopolysaccharide [34]. Each mutation was tested to determine its effect on growth rate and decay rate (stability) under the heat-shock conditions. All four of the heat-shock mutations significantly (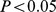) increased growth rate, 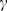, over the ancestor, ID8 (Figure 2; Table 2). Mutations T2, T3, and T4 significantly (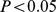) improved decay rate, 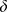, relative to the ancestor (Figure 2; Table 2). Although the T1 mutation improved the decay rate from 116.54 to 112.90, this change was not statistically significant (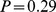). The combined effects of the growth rate and decay rate yield an overall fitness, 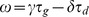, and all four of the heat-shock mutations significantly (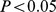) increased their overall fitness, 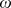, relative to the ancestor (Figure 2; Table 2).

**Table 1:** Mutations beneficial to ID8 under two selection regimes.

Label	Selection	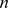	Protein function	Protein name	Aa position	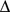 Aa	Nuc position	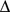 Nuc
T1	80°C	1	coat	F	5	T 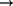 A	2578	A 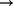 G
T2	80°C	1	coat	F	355	P 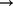 S	3628	C 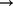 T
T3	80°C	1	spike	G	168	R 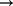 C	4484	C 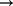 T
T4	80°C	2	spike	G	38	R 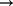 C	4094	C 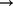 T
P1	pH 1.5	2	coat	F	77	I 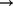 T	2792	T 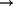 C
P2	pH 1.5	1	spike	G	65	T 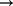 A	4175	A 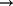 G
P3	pH 1.5	2	pilot	H	71	A 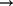 V	4738	C 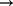 T

The seven beneficial mutations identified in this study affected three different viral structural proteins: the major coat protein F, the spike protein G, and the pilot protein H. Positions are based on the published genome sequence of ID8 (GenBank accession # DQ079898). Nuc, nucleotide; *n*, number of lineages with mutation; 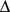
Nuc, nucleotide change; Aa, amino acid; 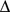
Aa, amino-acid change.

**Table 2:** The effects of mutations under heat-shock and low-pH conditions.

Mutation	Assay	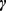	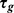	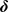	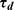	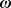
ID8	HS	13.64	1	116.54	0.083	3.93
T1	HS	16.37*	1	112.90NS	0.083	6.96*
T2	HS	14.73*	1	75.71*	0.083	8.42*
T3	HS	16.69*	1	78.95*	0.083	10.11*
T4	HS	15.21*	1	65.36*	0.083	9.76*
P1	HS	16.48*	1	159.55*	0.083	3.18ns
P2	HS	17.69*	1	133.99+	0.083	6.52*
P3	HS	14.65+	1	124.32ns	0.083	4.29+
ID8	pH	14.16	1	117.52	0.05	8.28
P1	pH	17.64*	1	104.24ns	0.05	10.36*
P2	pH	16.68*	1	98.83+	0.05	9.70*
P3	pH	15.44*	1	76.14*	0.05	7.75*
T1	pH	15.87*	1	145.17*	0.05	12.43ns
T2	pH	15.64*	1	105.55ns	0.05	11.73*
T3	pH	16.30*	1	132.04ns	0.05	11.63+
T4	pH	14.17ns	1	128.49ns	0.05	8.61ns

Selection for increased growth rate, 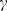, occurs during the growth phase of each transfer which lasts for time, represented as fraction of an hour, 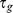. In the absence of host organisms, viral particles decay at rate 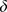 for a time, 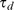. During this second stage of the regime, we subjected virus populations to either extreme heat (80°C) or low pH (1.5). Fitness, 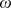, is measured as a combination of the growth and decay rates and the time spent under each condition, 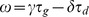. HS, heat-shock; pH, pH-shock. P values result from pairwise comparisons with sequential Bonferroni corrections for multiple comparisons to determine differences from the ancestor, ID8 (NS 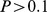; + 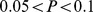; * 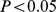).

**Figure 3:**
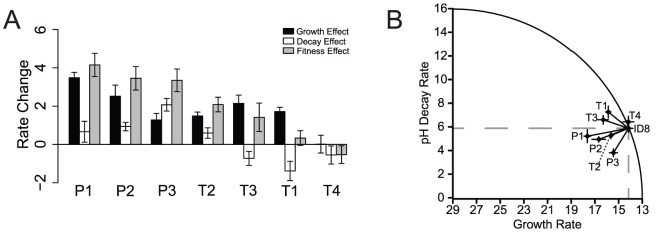
Low-pH-stability and growth-rate effects of all seven mutations. (A) For visual clarity, we plotted 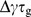, 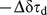, and 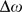 for growth, decay, and fitness effects. Overall, under the pH-shock conditions, the three unique low-pH mutations improved growth rate, decay rate, and overall fitness relative to the ancestor genotype, ID8. The four unique heat-shock mutations were variable in their effects relative to the ancestor. Mutation T2 improved both growth rate and decay rate relative to the ancestor; mutations T1 and T3 improved growth rate and worsened decay rate relative to the ancestor; mutation T4 growth rate and decay rate did not differ statistically from the ancestor. (B) The circle plots assume an optimal decay rate of zero and an optimal growth rate of 29 (the highest we have ever seen for this family of phages) [39]. Inside the curve is beneficial overall. Inside the dotted lines is beneficial for both traits. All of the low-pH mutations were beneficial for both traits, and one heat-shock mutation was beneficial for both traits relative to the ancestor under pH-shock conditions.

To determine whether the synergistic relationship between growth rate and decay rate is specific to heat-shock, we incorporated a second harsh selective pressure, low pH. We adapted five lineages from the same ancestral genotype to alternating selection for increased growth rates and selection for low pH tolerance in the absence of growth until a single mutation fixed in the population, which fixed within 11 transfers. We employed full-genome sequencing for each population to identify the first fixed mutation, and plaque isolates from the evolved populations were sequence-confirmed to ensure that only a single mutation was tested. We found three unique, first-step low-pH mutations in populations subjected to extreme pH-shock experiments (Table 1). Mutation P1, identified in two replicate lineages, was in the F gene encoding for the viral coat protein; mutation P2, identified in one lineage, was in the G gene encoding for the spike protein; mutation P3, identified in two replicate lineages, was in the H gene encoding for the pilot protein (Table 1). The protein encoded by the H gene is thought to form a tube to deliver viral genome DNA across the host periplasmic space and into the cytoplasm [35]. Each mutation was tested to determine its effect on growth rate and decay rate (stability) under the pH-shock conditions. All three of the pH-stability mutations significantly (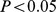) increased growth rate, 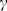, over the ancestor, ID8 (Figure 3; Table 2). Mutation P3 significantly (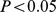) decreased decay, 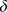, rate relative to the ancestor (Figure 3; Table 2); although mutations P1 and P2 reduced decay rate relative to the ancestor, the change was marginally significant for P2 (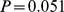) and nonsignificant for P1 (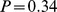; Table 2). The combined effects of the growth rate and decay rate yield an overall fitness, 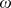, and all three of the pH-stability mutations significantly (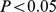) increased their overall fitness, 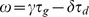, relative to the ancestor (Figure 3; Table 2). We found no pleiotropic costs of capsid stability on growth rate when exposed to pH-shock conditions.

Other studies comparing growth rate and decay rate have detected pleiotropic tradeoffs. De Paepe and Taddei compared lytic phages of *E. coli* and suggested that changes in capsid structure resulted in a tradeoff between survival and reproduction [23]. They proposed increased stability of the capsid enabled the virus to better package its DNA, but the dense packaging of DNA resulted in a slower replication rate. However, this study identified growth rate and decay rate in a variety of phages, but did not actively select for either trait. Studies exposing bacteriophages 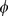
X174 and ID11 to moderate temperatures (37°C and 41.5°C) in *E. coli* hosts resulted in changes in viral capsid proteins that were likely stabilizing and came with a growth-rate cost [7], [8]. However, these studies did not de-couple growth rates and decay rates nor did they specifically select for both traits simultaneously. Dessau et al. [36] found a single antagonistically pleiotropic mutation in bacteriophage 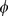
6 that resulted in a tradeoff between survival and reproduction after exposing evolving populations to a heat-shock selection. Our results may differ from this study because our two-stage selective regime fluctuated rapidly between selection for growth rate and decay rate thereby selecting on multiple fitness components, whereas the 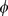
6 study imposed strong selection for heat resistance, a single fitness component. Additionally, our study found mutations influencing the interfacial bindings between proteins in the viral capsid, whereas Dessau et al. [36] found a single mutation influencing an enzyme. Contrary to these studies and our hypotheses, our populations exhibited no pleiotropic costs of capsid stability on growth rate when exposed to the heat-shock and pH-shock conditions, which imposed nearly simultaneous selection on both traits. Other recent studies found lack of tradeoffs when looking for evidence for antagonistic pleiotropy. Goldman and Travisano [37] exposed *E. coli* populations to ultraviolet radiation and found that increased survival did not come with a fitness cost, despite the fact that survival was negatively impacted in other harsh conditions. Leiby and Marx [38] readdressed the original findings that *E. coli* populations evolving high function on one resource for 20,000 generations gained a tradeoff with response to novel resources. By improving their assay method, they found that populations gained novel function for other resources suggesting that loss of function does not result as an inevitable consequence of adaptive tradeoffs, but instead could be due to the accumulation of disabling mutations in unused portions of the genome [38]. Antagonistic pleiotropy may not be as ubiquitous as previously thought, particularly when selective pressures act on multiple traits simultaneously.

### Tradeoffs across conditions

We tested whether mutations gained under one selective condition would be beneficial or detrimental when exposed to the other. Populations performed significantly better when assayed under the same condition in which the mutation was initially fixed compared to the alternate condition. As expected, growth rates for the extreme-heat mutations and low-pH mutations did not differ across assays (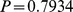). The effects under heat-shock conditions showed that the heat-shock mutants performed significantly better than the low-pH selected mutants (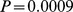), having more favorable decay rates. Likewise, under low-pH assay conditions, low-pH mutants performed significantly better than heat-shock mutants (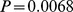). Fitness, 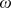, was significantly better for heat-shock mutants under heat-shock conditions (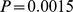), and pH-stable mutants were more fit under low-pH conditions than heat-shock mutants (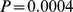). Additionally, none of the same mutations fixed between the heat-shock lineages and the pH-shock lineages (Table 1). We tested for changes in decay rates relative to the ancestor across environmental conditions and detected evidence for tradeoffs for some mutations. When exposed to heat-shock conditions, the pH mutation P1 worsened its decay rate relative to the ancestor (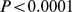), indicating a tradeoff between growth rate and decay rate. Although mutations P2 and P3 also worsened decay rate relative to the ancestor, these differences were marginally significant for P2 (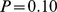) and nonsignificant for P3 (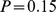). When exposed to pH-shock conditions, the T1 heat-shock mutation worsened its decay rate relative to the ancestor (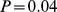), indicating a tradeoff between growth rate and decay rate. Although mutations T3 and T4 also worsened decay rate relative to the ancestor, these differences were nonsignificant (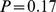 and 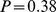, respectively). Our results indicate that our two selective regimes selected for different biophysical properties specific to the type of environmental selective pressure, and that tradeoffs arose when mutations were tested in the other extreme environmental condition. Selection for increased capsid stability under one extreme environmental condition can come with a tradeoff between growth rate and decay rate when exposed to the other condition. These results are consistent with other studies showing that improved fitness as a result of selection on one trait does not translate to improved fitness for traits not directly under selection [4], [7]–[9].

### Selection finds tradeoffs when it is not looking for payoffs

**Figure 4:**
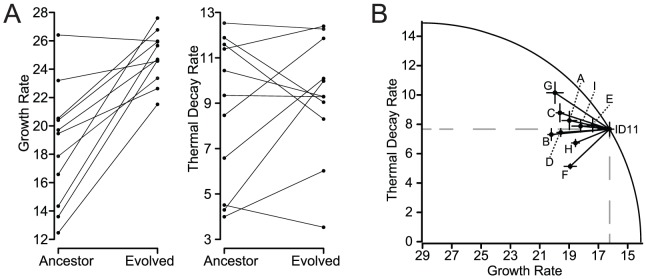
Selection for increased growth rate yields both tradeoffs and payoffs. We measured the effects on thermal stability of microvirid genotypes selected strictly for increased growth rate. (A) The 11 long-term adaptation lineages for eight different microvirid genotypes from Rokyta et al. [39] all increased or maintained their ancestral growth rates under selection. Decay rate at 80°C, which was a trait not under selection, increased in some lineages and decreased in others, but had a mean effect not significantly different from zero. (B) The nine single mutations that increased growth rate for the microvirid phage ID11 identified by [40] similarly showed highly variable effects on death rate at 80°C. Some mutations exhibit a tradeoff between growth rate and decay rate, and others exhibit a payoff. The plot assumes that the optimal combination of phenotypes is a decay rate of zero and a growth rate of 29, which was the highest value observed by Rokyta et al. [39] for growth-rate adaptation of microvirids. Mutations inside the curve were beneficial under the two-stage selection for thermal stability.

Simultaneous selection for increased growth rate at 37°C and decreased decay rate at 80°C or pH 1.5 revealed individual mutations that were beneficial to both traits, or synergistic pleiotropy, but such selection gives us a view of the possible variation that is biased toward such mutations if they exist. To determine the unbiased distribution of pleiotropic effects, we would need to select on each trait alone and measure the effects of fixed variants on the other trait. Such selection is impossible for decay rates alone, because the viral populations must be replenished by growth under some conditions, thereby selecting for growth properties. Selection for improved growth rates at 37°C is, however, possible and has been accomplished for numerous strains of microvirids. Rokyta et al. [39] selected 11 lineages started from eight genotypes, including two lineages of ID8, for increased growth rate until growth rate stopped improving, and Rokyta et al. [40] selected 20 lineages from the same ancestral genotype (ID11) and identified nine unique mutations that individually increased growth rate. We measured growth rates and decay rates under heat-shock conditions for the starting and ending genotypes of Rokyta et al. [39] (Figure 4). The ancestral and evolved forms differed by up to nine mutations. The average effect on growth was, as expected, significantly positive (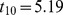, 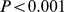) with an average improvement of 6.25 doublings per hour. The average pleiotropic effect on decay rate at 80°C was not significantly different from zero (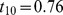, 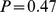) with a mean increase in decay rate of 7.66 doublings per hour or 0.64 doublings per five minutes (the time frame used in our selection experiments). The pleiotropic effects on decay rate ranged from a decrease in decay rate of 3.29 to an increase of 5.68 doublings per five minutes. For the nine growth-rate mutations for ID11 identified by Rokyta et al. [40], the average growth rate effect was significantly positive (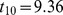, 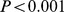) with a mean of 2.81 doublings per hour. The pleiotropic effect on thermal decay rate was not significantly different from zero (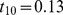, 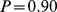) with a decrease of 0.06 doublings per five minutes. The decay-rate effects ranged from a decrease of 2.54 to an increase of 2.48 doublings per five minutes. We found that for selection on growth rate alone, nearly half of the mutations showed a payoff consistent with our experiments above, but the other half exhibited a tradeoff. Other studies have found variable pleiotropic effects with response to exposure to a novel trait or function [41], [42]. For example, Travisano et al. [41] found that some *E. coli* populations evolved for 2,000 generations in glucose had improved fitness in maltose, but others had reduced fitness. The variability observed was due to mutations that differed in their pleiotropic effects on fitness in maltose. A similar study determined pleiotropic effects to a range of novel resources and found that most pleiotropic effects were synergistic, but mutations yielding antagonistic effects also arose [42]. Mutations with antagonistically pleiotropic effects can arise when selection is not looking for a payoff on multiple traits.

We hypothesized that constraints exist in the ability to fix a mutation beneficial for both survival and reproduction. Such constraints may be avoided in systems with higher mutation rates because mutation supply is not limited, allowing populations with greater genetic variation more evolvablility and a greater capacity to respond to environmental change [43]–[46]. Single-stranded DNA bacteriophages have mutation rates of 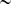 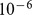 mutations per base per round of replication, which is greater than other DNA-based organisms with rates as low as 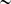 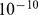 [47]. Under our two-stage selection with high mutation rates, selection easily found synergistically beneficial mutations for both reproduction and survival, whereas dsDNA systems with lower mutation rates may not find rare, synergistically pleiotropic mutations.

### The biophysical mechanisms of stability increases

Because of the inherent complexity of viral capsids, it is challenging to experimentally quantify how mutations affect viral stabilities and to decipher the underlying nature of each thermally selected spot in the ancestral virus. Since the geometry of icosahedral capsids suggest that stability should depend largely, if not entirely, on binding strength between capsid subunits, mutations were assessed for their effects on binding strength between the major-capsid and major-spike proteins. We employed a molecular-dynamics simulation method, the orthogonal space tempering (OST) algorithm [48], to computationally assess the viral capsid stability changes in response to three beneficial heat-shock mutations (T2: Pro-F355-Ser; T3: G: Arg-G168-Cys; T4: Arg-G38-Cys; Table 1), which occur either at the coat-coat interfaces (T2) or at the spike-coat interfaces (T3 and T4), and analyzed the intrinsic contribution that each of the original ancestor residues makes to the capsid stability (Figure S1). Mutation T1 was not simulated because it resides in a disordered terminus with no structural information [49]. We hypothesized that the simulations would reveal enhanced binding affinities between capsid protein subunits of the mutations that decrease decay rate relative to the ancestral virus during the empirical procedures, providing support for the use of biophysical simulation strategies to predict the effects of mutations on protein and macromolecular stability.

**Figure 5:**
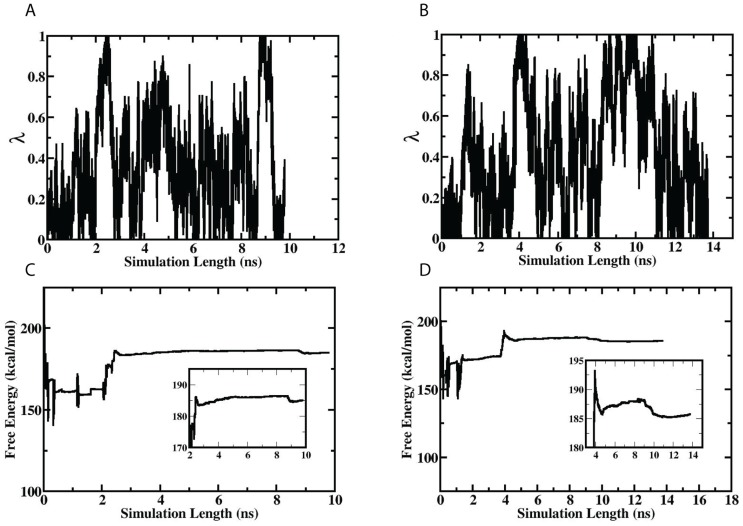
The time-dependent scaling parameter changes and free-energy convergences of a representative set of IBC simulations. (A,C) The left column shows the results of the simulation on the 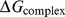
(G:Arg38) calculation and (B,D) the right column shows the results of the simulation on the 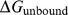
(G:Arg38) calculations. (A,B) The upper row shows the time-dependent scaling parameter changes, and (C,D) the lower row shows the convergence behaviors of the free-energy estimations.

**Figure 6:**
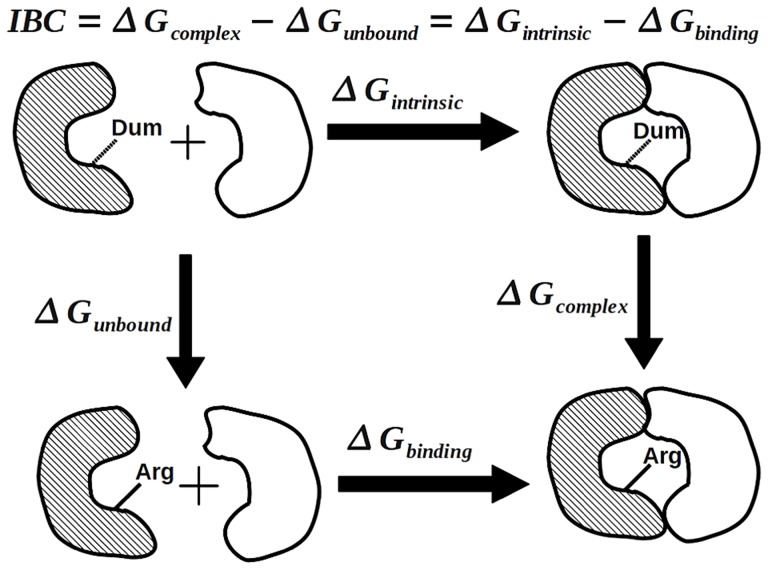
The thermodynamic cycle for the calculation of the intrinsic binding contribution of a protein-protein interfacial residue. An idealized dummy residue was employed as the reference to evaluate the intrinsic contribution of a target residue (for instance Arg) to the binding strength of the two interacting proteins. The intrinsic binding contribution (IBC) of this Arg residue is defined as 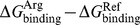. To evaluate the IBC, two alchemical calculations are performed respectively to calculate 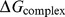 and 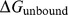.

The simulation results revealed that all three beneficial mutations significantly enhance the affinities of the corresponding protein-protein interfacial bindings and therefore greatly improve the capsid stability. In a representative set of calculations on 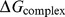
(G:Arg38) and 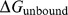
(G:Arg38) (Figure 5), multiple alchemical transitions between the real chemical state 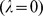 and the dummy state 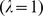 were realized within about 15 nanoseconds (ns). The simulation timescales were much shorter than realistic biological timescales but were, however, sufficient for the OST enhanced sampling algorithm to obtain confident free-energy convergences [48], [50], [51]. The first one-way trips were complete within 3 ns for the simulation on the complex and 5 ns for the simulation on the unbound environment. The long-waiting time between the revisits of the dummy states (7 ns for the simulation on the complex and 4 ns for the simulation on the unbound structure) indicate that these alchemical transitions were coupled with slow conformational changes, which are usually forbiddingly challenging for the other methods to efficiently explore. After the simulations sampled both of the end states, the estimated free-energy changes arrived at their converging phases as suggested by their time-dependent fluctuating behaviors. In a representative simulation set, we estimated 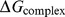
(G:Arg38) to be 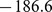 kcal/mol and 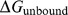
(G:Arg38) to be 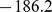 kcal/mol; thus the intrinsic binding contribution (IBC) of the G: Arg38 residue can be evaluated to be 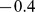 kcal/mol, which is a fairly modest value (Table 3; Figure 6). All the other free-energy simulations display similar sampling and convergence behaviors (Table 3; Figure S2–S6).

**Table 3:** The protein-protein interfacial binding changes and their contributing components.

Mutation	Residue	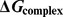	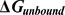	IBC	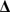 IBC	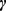	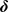	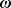
T2	Ancestor: Pro	24.6	22.9	1.7	−9.2	13.64	116.54	3.93
	Mutant: Ser	9.5	16.9	−7.4		14.73	75.71	8.42
T3	Ancestor: Arg	−186.6	−186.2	−0.4	−4.5	13.64	116.54	3.93
	Mutant: Cys	7.7	12.6	−4.9		16.69	78.95	10.11
T4	Ancestor: Arg	−185.6	−185.6	0.0	−8.2	13.64	116.54	3.93
	Mutant: Cys	11.7	19.9	−8.2		15.21	65.36	9.76

IBC, intrinsic binding contribution; 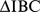, change in intrinsic binding contribution from ancestral to mutant residue; 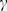, growth rate; 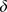, decay rate; 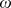, fitness.

The ancestral residues (F: Pro355; G: Arg168; G: Arg38) at the beneficial mutation sites displayed either unfavorable (1.7 kcal/mol) or very modest (0.0 kcal/mol and 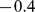 kcal/mol) intrinsic contributions to the protein-protein interfacial binding and were therefore not irreplaceable in terms of virus capsid stability (Table 3). Even considering possible numerical error, no significant intrinsic binding contribution is likely to be displayed by these ancestral amino acids. Note that Pro and Arg possess different chemical natures: Pro is nonpolar/hydrophobic and Arg is very hydrophilic; apparently each resides in a unique non-optimized protein-complex environment (Figure S7). In contrast, all the selected beneficial mutant residues were inherently suitable for the protein-protein interfacial regions by displaying very favorable (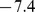 kcal/mol, 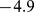 kcal/mol, 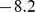 kcal/mol) IBC values. In terms of their chemical nature, a polar Ser residue was selected to replace Pro at the F355 position and hydrophobic Cys residues were selected to replace Arg residues at the G38 and G168 spots. Consequently, all three beneficial mutations significantly enhanced the affinities of the corresponding protein-protein interfacial bindings by 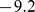 kcal/mol, 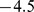 kcal/mol, and 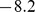 kcal/mol respectively and therefore greatly improved the capsid stability (Table 3; Figure S2–S6). Furthermore, each mature capsid has 60 copies of both the F and G proteins, meaning that these binding energies were effectively much larger in the overall capsid structure.

The thermal selection experiments were carried out at two temperatures: 37°C and 80°C. For beneficial heat-shock mutations, binding affinity increases are unlikely to be associated with large entropy decrease; for instance, favorable Arg-to-Cys mutations are expected to cause increases of protein-protein binding entropy. Our results and conclusion derived from the simulations at 26.85°C should be transferable for the 37°C and 80°C conditions, unless the virus capsid undergoes structural phase transition between these temperatures.

Our results suggest that null spots exist in the ancestor virus that can be readily selected by high temperature selection without a need of tradeoff. We demonstrated that biophysical simulation strategies, as used here, can be an effective tool to predict the effects of mutations on protein stability. Studies have also shown that more stable, compact structures can resist denaturation, allowing proteins to evolve to carry out their functions or at least remain folded under extreme environmental conditions [52]. These stable backgrounds can better tolerate the effects of deleterious mutations that alter protein function [53]–[55]. Simultaneous selection for both growth rate and capsid stability can result in viral populations that not only withstand extreme environmental conditions, but can also resist the impact of deleterious mutations later in the adaptive walk, thereby promoting evolvability of the populations [54], [56], [57]. Our work shows the feasibility of using computational models as a predictive tool for the effects of mutations on protein stability. Ultimately, computational models similar to those used in this study could be developed to predict a population's adaptive evolution. Moreover, all-atom simulation-based analyses allow for a greater understanding of the relationship between mutational effects and viral capsid structures. Future empirical work and computational modeling to determine and predict the threshold of the functional range that a viral capsid can endure could impact many research areas including protein engineering and disease transmission.

## Materials and Methods

### Heat-shock selection

Serial transfers were performed similar to a previously described method [40] with modifications. All replicate lineages were started from individual plaques isolated from a single ancestral genotype (ID8a0) [39] and passaged through serial transfers under a two-stage selection regime until a single beneficial mutation fixed in the population. A culture of host cells (*E. coli* strain C) were grown to a density of 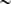 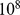 cells per ml in 10 ml of Lysogeny Broth (10 g Tryptone, 10 g NaCl, 5 g yeast extract per liter, supplemented with 2 mM CaCl_2_) within a 125 ml Erlenmeyer flask at 37°C in an orbital shaking water bath set to 200 RPM. The culture was inoculated with 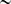 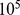 phage and allowed to propagate for 60 minutes, reaching a density of 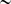 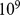
–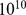 pfu/ml. This growth phase was halted by taking an aliquot of the culture and exposing it to CHCl_3_, to stop cell growth, followed by centrifugation to remove the cellular debris. One milliliter of the phage–laden supernatant was separated into two 0.65 ml microcentrifuge tubes at 500 *µ*l each. The two tubes were placed into an ice bath for five minutes to normalize the starting temperature for the subsequent heat-shock. After cold exposure, the 0.65 ml tubes were transferred to a Perkin Elmer Cetus DNA thermal cycler set to 80°C and incubated for five minutes, and then transferred back to the ice bath for five minutes to reduce the temperature, stopping the heat shock. An appropriate aliquot was then transferred to the subsequent host culture and allowed to grow again. Population sizes were monitored by plating on agar plates at three points for each growth-death cycle; initial concentration prior to growth, concentration after growth, and concentration after heat shock. Population change rates were calculated on a 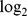 scale resulting in values of population doublings/halvings per hour.

### pH-shock selection

Serial transfers were performed under pH stress conditions similar to the heat-shock methods. A culture of *E.coli* strain C were grown to a density of 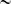 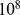 cfu/ml at 37°C shaking at 200 RPM in an orbital waterbath, and inoculated with 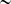 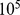 phage and grown for 60 minutes. After the growth phase, aliquots were removed, exposed to CHCl_3_, and centrifuged to remove cellular debris. One milliliter of the phage containing supernatant was transferred to a sterile glass test tube at room temperature. The pH was lowered to 1.5 with 0.5 M HCl for three minutes and brought back to pH 7 with 0.5 M NaOH. An aliquot of the pH shocked population was transferred to a new host culture and allowed to grow, repeating the serial transfer process. Population densities were measured at three points for each transfer; before host inoculation, after the growth phase, and after pH shock. Population size change rates were calculated on a 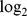 scale as with the heat-shock procedure.

### Sequencing

We sequenced the entire genome of the final population of each lineage. Whole population sequencing allows detection of mutations that have fixed or reached high frequency. For each lineage, we identified only a single mutation. We then sequenced a plaque isolate from each final population per lineage. The sequence-confirmed isolates were used for all fitness assays.

### Fitness assays

We measured fitness in our selective environment by calculating the population change rates on a 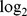 scale resulting in values of population doublings/halvings per hour. Fitness was measured in conditions identical to our selective environment with isolates no more than a week old. Selection for increased growth rate, 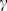, occurs during the growth phase of each transfer which lasts for time 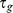. In the absence of host organisms, viral particles decay at rate 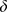 for a time, 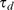, under harsh conditions until permissive hosts are present again. During this second stage of the regime, we subjected virus populations to either extreme heat (80°C) or low pH (1.5). Fitness, 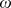, is measured as a combination of the growth and decay rates and the time spent under each condition, 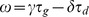, following the notation laid out by Handel et al. [20]. Fitness measures were replicated at least 5 times for each isolate.

### Computational model design and setup

We focused our analysis on three beneficial mutations, including T2 (F: Pro355Ser), T3 (G: Arg168Cys), and T4 (G: Arg38Cys)]. Mutation T1 was not simulated because it resides in a disordered terminus with no structural information [49]. The T2 mutation occurs at the coat-coat interfaces and the T3 and T4 mutations occur at the spike-coat interfaces. Specifically, the alchemical free-energy simulation scheme [58]–[61] was employed to quantify the intrinsic contribution of each involved residue to the stability of the target structure constructs. For instance, to analyze the intrinsic binding contribution (IBC) of the ancestor residue G:Arg168 to the corresponding spike-coat complex formation, we applied the thermodynamic cycle in Figure 6, where a conceptual amino acid with a dummy side-chain was used as the reference and the IBC could be evaluated by means of calculating the free-energy differences between the reference dummy state and the target Arg state respectively in the complex structural construct and the unbound form of the spike, i.e. 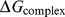
(G:Arg168) and 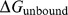
(G:Arg168). In correspondence, the protein-protein interfacial binding change due to the T3 mutation can be estimated by the difference between IBC(G: Arg168) and IBC(G: Cys168).

To build the atomistic simulation models, an X-ray crystal structure of bacteriophage G4 (PDB ID: 1GFF) [49] was modified to match the sequence of the target bacteriophage ID8. For each free-energy calculation on 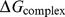, the model comprised of all of the atoms of the ID8 bacteriophage spike-coat pentameric protein complex within a radius of 40 Å around the 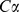 atom of the target spot and a truncated-octahedral box of water molecules that solvate the protein atoms. The representative model for the 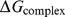
(G:Arg168) calculation is illustrated in Figure 6. For each free-energy calculation on 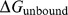, the model was built in the same way as the above except that only the atoms of the unbound form of the target protein are included. In addition, potassium and chloride ions were added to neutralize the systems with the ionic strength adjusted to be 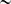
0.15 mol/L. The sizes of the models range from 89
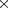
89
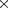
89 Å [60] to 99
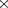
99
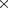
99 Å which have about 13,000 to 20,000 water molecules. The protein and ion molecules were treated with the CHARMM27 model [62] and water molecules were described by the TIP3P model [63]. For all the simulations, the particle mesh ewald (PME) method [64] was applied to describe long-range columbic interactions and short range interactions were switched off at 10 Å.

Langevin dynamics were used to maintain the constant temperature, which is set at 26.85°C; the propagation time-step was set as 1 femtosecond (fs) and the friction constant was set to 100 picosecond (ps)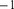. To maintain the structural integrity, the positions of the protein atoms beyond a radius of 30 Å of the center were fixed. The alchemical free-energy calculations were performed based on a modified orthogonal space tempering (OST) algorithm [48], [50], [51], which in comparison with the original OST method includes an additional treatment on weakly-coupled fluctuations. In these simulations, a hybrid energy function 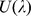 was used; 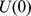 represents the energy function of the real chemical state and 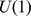 represents the energy function of the reference dummy state (Figure S2). In these alchemical transitions, the van der Waal and electrostatic energy terms of the atoms associated with the dummy states were treated with a 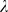-dependent soft-core potential [65]. For the alchemical changes involving non-Pro residues, the bond and angle terms of the dummy atoms remained the same as the corresponding ones at the real chemical state; for the alchemical change involving the Pro residue, the dihedral terms of the dummy atoms were also kept the same as the corresponding ones at the real chemical state. In OST, the scaling parameter 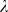 was treated as a one-dimension particle that can dynamically moves between 0 and 1 (the two end states), to collect samples for free-energy estimations. To overcome the time-scale limitation, the OST method selectively enlarged fluctuations of critical environments that had strong and weak coupling with chemical state 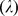 transitions. It should be specially noted that the directly computed free-energy difference 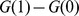 had an opposite sign from the IBC defined in Figure 6. The signs of the free-energy values in Table 3 are summarized in consistence with the description in Figure 6.

### Statistical analyses

We used mixed linear models for each shock assay to assess the dependence of growth rate, decay rate and overall fitness on the fixed predictor variable evolutionary history (ancestor versus mutant) and the random predictor variable phage population. Pairwise comparisons and sequential Bonferroni corrections for multiple comparisons were used to determine growth rate, decay rate, and overall fitness differences from the ancestor virus (PROC MIXED, SAS Institute 2009).

## Supporting Information

Figure S1A representative model setup for the calculation of 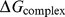 (G:Arg168). The overall structure of the ID8 bacteriophage spike-coat pentameric protein complex and the target residue (G: Arg168), which is located at the center, are highlighted in red. In the simulation model, the atoms beyond a radius of 40 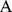 around the center are cut. In the model, the major spike proteins G are colored in blue and the major coat proteins F are colored in green.(EPS)

Figure S2The time-dependent scaling parameter changes and free-energy convergences of the G:Cys38 set of IBC simulations. (A,C) The left column shows the results of the simulation on the 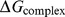 (G:Cys38) calculation and (B,D) the right column shows the results of the simulation on the 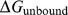 (G:Cys38) calculations. (A,B) The upper row shows the time-dependent scaling parameter changes, and (C,D) the lower row shows the convergence behaviors of the free-energy estimations.(EPS)

Figure S3The time-dependent scaling parameter changes and free-energy convergences of the G:Arg168 set of IBC simulations. (A,C) The left column shows the results of the simulation on the 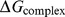 (G:Arg168) calculation and (B,D) the right column shows the results of the simulation on the 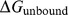 (G:Arg168) calculations. (A,B) The upper row shows the time-dependent scaling parameter changes, and (C,D) the lower row shows the convergence behaviors of the free-energy estimations.(EPS)

Figure S4The time-dependent scaling parameter changes and free-energy convergences of the G:Cys168 set of IBC simulations. (A,C) The left column shows the results of the simulation on the 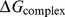 (G:Cys168) calculation and (B,D) the right column shows the results of the simulation on the 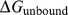 (G:Cys168) calculations. (A,B) The upper row shows the time-dependent scaling parameter changes, and (C,D) the lower row shows the convergence behaviors of the free-energy estimations.(EPS)

Figure S5The time-dependent scaling parameter changes and free-energy convergences of the F:Pro355 set of IBC simulations. (A,C) The left column shows the results of the simulation on the 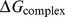 (F:Pro355) calculation and (B,D) the right column shows the results of the simulation on the 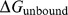 (F:Pro355) calculations. (A,B) The upper row shows the time-dependent scaling parameter changes, and (C,D) the lower row shows the convergence behaviors of the free-energy estimations.(EPS)

Figure S6The time-dependent scaling parameter changes and free-energy convergences of the F:ser355 set of IBC simulations. (A,C) The left column shows the results of the simulation on the 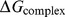 (F:Ser355) calculation and (B,D) the right column shows the results of the simulation on the 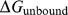 (F:Ser355) calculations. (A,B) The upper row shows the time-dependent scaling parameter changes, and (C,D) the lower row shows the convergence behaviors of the free-energy estimations.(EPS)

Figure S7 The representative alchemical changes. (A) The representative alchemical change from a non-Pro residue to its reference state, in which all the side chain atoms are converted to the corresponding dummy atoms. (B) The alchemical change from a Pro residue to its reference state; here a hybrid-topology approach is employed to convert a real Pro side chain/a dummy hydrogen to a dummy Pro/a real hydrogen. The red regions stand for the dummy atoms and the blue region highlights the atoms which partial charges and atom types vary between two states.
(EPS)
